# Role-Based Cyberbullying Situations: Cybervictims, Cyberaggressors and Cyberbystanders

**DOI:** 10.3390/ijerph18168669

**Published:** 2021-08-17

**Authors:** Víctor González-Calatayud, María Paz Prendes Espinosa

**Affiliations:** 1 Department of Statistics, Mathematics and Computer Science, Miguel Hernández University, 03202 Elche, Spain; 2 Department of Didactics and School Organization, University of Murcia, 30100 Murcia, Spain; pazprend@um.es

**Keywords:** cyberbullying, cybervictim, ICT, secondary education

## Abstract

The use of information and communication technology (ICT) has led to new risks, and among them is cyberbullying. It is important to be aware of the prevalence of cyberbullying in order to design intervention plans based on real contexts. Studies, however, vary widely in the data they report. These discrepancies may be due to differences in measurement. The main aim of our study, conducted in the Region of Murcia (Spain), was to ascertain the prevalence of cyberbullying in the three roles involved: victim, perpetrator and bystander. A descriptive, correlational and quantitative study was conducted using a “Cyberbullying: peer harassment screening”questionnaire to collect data. The representative sample comprised 950 students aged between 11 and 18 years (M = 13.93, SD = 1.35). The data showed that 72.1% of the participants had been involved in one or more cyberbullying situation (as victim, aggressor and/or bystander) in the previous year. Specifically, 49.3% had been cybervictims, 23.3% cyberaggressors and 62.3% cyberbystanders. The study provides detailed information about the prevalence of cyberbullying in the Region of Murcia and enables comparisons of the three roles involved. The data point to the need to promote active prevention and psychoeducational intervention strategies.

## 1. Introduction

The new possibilities afforded by the use of information and communication technology (ICT) for communication between persons mean that we can now be in contact, form groups, organize ourselves and exchange knowledge as never before [1]. However, the fact that our lives are increasingly linked to technology means that real world problematic situations have spilled over into the virtual world. One example is school bullying—ICT has provided a platform for new forms of bullying, known as cyberbullying. In its broadest sense, cyberbullying is any behavior that is performed using technology, particularly the internet, by an individual or group that involves repeatedly sending aggressive messages or other similar actions with the intention of causing harm [2]. Like traditional bullying, cyberbullying can have long-term effects for its adolescent victims [3], with technology being one of the most important risk factors [4].

If we focus on the data regarding the prevalence of this problem, we can see that interest in this phenomenon has increased in recent years, with more and more data becoming available since the first study in the US in 2000 [5]. Studies to date have varied in their understanding of the problem and how to measure it, but most suggest that 20–50% of adolescents have had at least one experience of cyberbullying and that the number of cybervictims is on the rise [2,6,7]. Many of these victims are also victims of traditional bullying, so there is a clear connection between both [8,9,10]. Furthermore, we find differences not only for victims, but also for aggressors [11,12], with differences in both gender and age.

A study by Garaigordobil [13] in the Basque Country reported that 30.2% of participants stated that they had endured one or more forms of cyberbullying in the past year, 15.5% said that they had perpetrated one or more of them and 65.1% declared that they had seen one or more of them. This study is noted as it used the same indicators as our study to evaluate cyberbullying. A recent systematic review of the literature on cyberbullying among teenagers in Spain [14] revealed that the prevalence rate in these studies is 26.65% (SD = 23.23), with a range from 78.31% (which includes occasional cases) to 4.6%. In terms of cyberbullies, the data show a rate of 24.64% (SD = 24.35), with a range from 56.5% to 2%.

Data also vary a great deal in recent national and international studies. Ortega-Barón, Buelga and Cava [15] found that 27.4% of subjects had been victims of cyberbullying in the previous year, of which, 20.5% had experienced moderate cyberbullying and 5.5% had experienced severe. Lee and Shin [16] reported that 34% of their sample had been involved in episodes of cyberbullying, either as victims (14.6%), aggressors (6.3%) or victims/aggressors (13.1%). Similarly, Herrera-López, Romera and Ortega-Ruiz [17] found that 10.7% had been involved as cybervictims, 2.5% as cyberaggressors and 5.5% as cyberaggressors/victims, with a total involvement of 18.7%.

Therefore, the data show large differences in the numbers of victims and aggressors. These differences may be due to how cyberbullying is conceived in terms of one of its fundamental characteristics—repetition. Some studies state the necessity to consider repetition as a key issue, as is done in traditional bullying [18,19,20,21]. Others, however, assert that, in the case of ICT, it is impossible to know how many times a video or website is seen, with the result that victims suffer humiliation repeatedly from a single episode [9,22,23,24,25].

Hence, the instruments used in different studies may include a single question that addresses the whole problem, or may be multi-item asking about various related behaviors. However, as Menesini and Nocentini [26] state, it is preferable to use a multi-item scale, given the complexity of the phenomenon and that the reliability and validity of these types of instruments tend to be better. We also found that, in most studies, researchers had created a new measurement, the psychometric parameters of which were often not included, and the measurement scales and periods of which could vary [6,27,28]. All of this makes it difficult to compare the phenomenon and see how it has evolved.

Regarding the assessment of cyberbullying using multi-item instruments, the main behaviors that participants in the different studies reportedly experienced, perpetrated or observed were, on one hand, sending insulting and frightening phone text messages [29,30] and, on the other, making anonymous phone calls aimed at scaring the recipients [13].

On the basis of earlier studies, the main aim of our research is, therefore, a descriptive quantitative study of the prevalence of cyberbullying in the Region of Murcia to ascertain the percentages of adolescents involved as cybervictims, cyberaggressors or cyberbystanders (population: 66,413; period of risk: one year). The results will allow us to compare our data with those of other countries and, especially, with other studies in Spain that use the same indicators, and to evaluate the seriousness of the situation and the need for prevention and intervention. Hence, we formulated four hypotheses to address in the analysis of the data: (1) over the last year, 50% of all students have been involved in episodes of cyberbullying, be it as victim, aggressor or bystander; (2) the prevalence of overall cybervictimization and cyberaggression (suffering or perpetrating the behaviors evaluated sometimes, often or always) will be between 1% and 10%, while severe cybervictimization and cyberaggression (suffering or perpetrating these behaviors often or always) will be below 2%; (3) there will be a high level of concordance between the cyberbullying prevalent in the three roles; and (4) there will be positive and significant correlations among the four cyberbullying indexes (cybervictimization, cyberaggression, cyberbystanders and cyberaggressive cybervictimization).

## 2. Materials and Methods

### 2.1. Participants

In order to get a representative sample for the Region of Murcia, we first ascertained how many students were in compulsory secondary education. The figure was 66,413. Therefore, for a confidence level of 95% and a sample error of 3.2% for a population variation of 0.50, a representative sample comprised 925 students. Stratified random sampling, specifically the school districts used by the educational authorities, was used to select the sample, which finally totaled 950 students from the Region of Murcia, aged between 11 and 18 years (M = 13.93, SD = 1.35), of which 479 were males (50.4%) and 471 females (49.6%). Students came from state secondary schools (64.6%) and private and semi-private schools (35.4%). Eleven schools were involved in the research.

### 2.2. Instrument

The “Cyberbullying: peer harassment screening” [30] instrument that was used complied with all the reliability and validity guarantees. The questionnaire comprised 45 items grouped into three factors related to the three related roles: victim, aggressor and bystander. Fifteen behaviors were assessed for each of the roles (e.g., sending offensive messages or stealing a password). Students read the behavior and indicated how often (never, in some cases, quite often or always) they had suffered from it, had perpetrated it or had observed it in the previous year. The instrument showed high internal reliability (α = 0.91), and Cronbach’s alpha was high for the three scales of the instrument: cybervictimization (α = 0.82), cyberaggression (α = 0.91) and cyberbystander (α = 0.87). Regarding reliability of the study sample, the instrument again showed high internal consistency (α = 0.91) and for all three scales: cybervictimization (α = 0.82), cyberaggression (α = 0.72) and cyberbystander (α = 0.93). The confirmatory factorial analysis found that the KMO value (0.94) and Bartlett’s test was significant (χ = 51,208.99, *p* < 0.001), confirming the three-factor structure that explained 40.15% of the variance [30]. Each factor was composed of 15 items.

### 2.3. Design and Procedure

This study used a descriptive, correlational and cross-sectional design, and a questionnaire to collect information. Three stages were followed: (1) the principals of the randomly selected schools were contacted by email or telephone. The project was explained to them and their collaboration was requested; (2) an interview was held with those who agreed to participate in order to explain the project in greater depth and to give them the informed consent sheet for the parents of the participating students; and (3) after receiving the informed consent from the parents, the questionnaire was administered on paper in a single session of 30 min. The study was approved by the ethics committee of the University of Murcia.

### 2.4. Data Analysis

Data analysis was conducting using SPSS 22.0, with a bilateral significance level of *p* < 0.05 for all of the study. The main techniques used were: frequency analysis and percentage for a preliminary descriptive analysis, contingency tables and Pearson’s chi-square statistic (χ^2^), and Pearson correlations to determine the existing relationship among the cyberbullying indexes. The database can be found at the following link: https://osf.io/bwq3j/?view_only=217aa5ce75704aa3a600393b38f578f2 (accessed on 22 June 2021).

For the analysis of each behavior, the criterion was to have experienced the same behavior at least once. Regarding the criterion for indicating whether a participant was at risk of cyberbullying, the one established in the tool’s manual was used.

## 3. Results

### 3.1. Students Involved in Cyberbyllying Episodes

Frequencies and percentages were calculated to identify those students who had been involved in cyberbullying episodes over the previous year and those who had not. The findings were that 72.1% (*n* = 685) said they had suffered, perpetrated and/or witnessed some of the cyberbullying behaviors being evaluated. Specifically, 49.3% (*n* = 468) stated that they had suffered one or more cyberbullying behaviors; 23.3% (*n* = 221) reported that they had perpetrated one or more cyberbullying behaviors; and 62.3% (*n* = 592) had witnessed these behaviors.

### 3.2. Prevalence of Cyberbullying According to the Valuations of Victims, Aggressors and Bystanders

First, we calculated the frequencies and percentages of participants who reported that, in the previous year, they had been cybervictims, cyberaggressors or cyberbystanders of the 15 cyberbullying behaviors under evaluation. The percentages were classified as moderate (sometimes) and severe (often and always), thus identifying the percentages of individuals participating in each condition (Table 1).

The analysis of cybervictim prevalence showed that, although the percentages were, in many cases, small for the behaviors assessed, the data did deserve consideration as some high percentages were also present. Overall, the data showed a range from 2.3% to 27.2% of participants who had experienced any of the 15 cyberbullying behaviors. If we focus on the severe cases (i.e., those who suffered these behaviors often or always), the percentages ranged from 0.3% to 4.9%. Elsewhere, the overall prevalence of cyberaggressors reporting that they had perpetrated one or more of these behaviors ranged from 0.5% to 12.5%. The analysis of severe cyberaggression revealed that between 0.1% and 1.1% stated that they had perpetrated some of these behaviors very frequently. Finally, the overall prevalence of cyberbystanders who stated that they had witnessed these behaviors ranged from 8.4% to 44.9%, while severe cases ranged from 2% to 13.1% for the behaviors evaluated.

### 3.3. Most Prevalent Behaviors Reported in the Three Roles

Table 1 shows the comparisons of the most prevalent behaviors in the three roles. There is a high convergence from the three perspectives (victim, perpetrator and bystander) for five of the behaviors: sending offensive, insulting messages by mobile phone or the internet (victim 27.2%, aggressor 12.5%, bystander 44.9%1); making anonymous calls to frighten and provoke fear (victim 22.2%, aggressor 9.3%, bystander 32.5%); slandering, saying things on the internet that are not true to smear the character, spreading harmful rumors (victim 17.3%, aggressor 1.8%, bystander 28.3%); making offensive, insulting phone calls or over the internet (victim 16.3%, aggressor 5.8%, bystander 35.3%); and blackmailing or threatening via messages or phone calls (victim 11.9%, aggressor 3.6%, bystander 23.2%).

### 3.4. Relation between Cybervictimization, Cyberaggression and Cyberbystanding

In order to analyze the total indexes obtained in the test of the four roles (cybervictimization, cyberaggression, cyberbystander and cyberaggressive-cybervictim), we obtained the bivariate partial correlation coefficients, controlling for the effect of sex and age. Table 2 shows the results indicating positive and significant correlations among the four total indexes, which suggests that cybervictims are more prone to using electronic means to perpetrate aggressive behaviors towards others and to score high on aggressive victimization.

**Figure 1:**
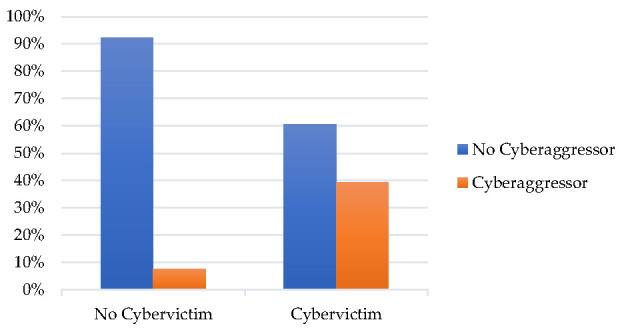
Percentage of cybervictims who are not cyberaggressors.

Additionally, a contingency table was constructed for those who stated that they had suffered one or more behaviors and for those who stated that they had perpetrated one or more episodes of cyberbullying in order to identify those who could be considered as pure victims and pure aggressors (Figure 1). Among the 49.2% (*n* = 468) stating that they had been cybervictims of one or more of the behaviors evaluated, 60.7% (*n* = 284) were pure victims who had never perpetrated episodes of online aggression towards others, while 39.3% (*n* = 184) were cybervictims and cyberaggressors over the previous year. Of the 23.3% of cyberaggressors who stated that they had perpetrated some episode of the behaviors, 16.7% (*n* = 37) were pure cyberaggressors who had not been the recipients of previous behaviors. Pearson’s chi-square confirmed the significant differences, χ^2^ = 133.16, *p* < 0.001.

## 4. Discussion

It should be noted that this research provides precise information about the current state of cyberbullying in the Region of Murcia for a representative sample aged between 11 and 18 years, and it enables us to compare the various roles of those involved (cybervictims, cyberaggressors and cyberbystanders). One of the main contributions of this study is its analysis of the prevalence of cyberbullying in the Region of Murcia.

First, the data provide an overall percentage of those involved in cyberbullying situations (be they victims, aggressors or bystanders)—72.1% in the previous year. Of these, 49.3% reported having suffered one or more instances of cyberbullying, 23.3% stated that they had perpetrated one or more instances and 62.3% indicated that they had witnessed one or more instances of the behaviors evaluated. Hence, Hypothesis 1 is confirmed, since the percentage of those involved (72.1%) clearly exceeds that hypothesized (50%), as do the percentages reported elsewhere [13,15,16,17].

Second, the results show that between 2.3% and 27.2% of subjects had suffered one or more of the behaviors evaluated (sometimes, often or always) and that between 0.3% and 4.9% had suffered these behaviors severely (often or always). Between 0.5% and 12.5% of cyberaggressors had perpetrated bullying episodes, with 0.1% to 1.1% doing so severely. The data show that a large percentage of students had observed some cyberbullying behavior in the previous year. Moreover, the data also show that some of these behaviors were observed continuously—often or always. The data confirm, but with caution, that our Hypothesis 2 is different from other studies that report cybervictimization levels of below 10% [3,12,17]. However, these discrepancies may, as previously mentioned, be due to the different instruments used, different scales and different periods of measurement, with some studies enquiring about the previous year, others about previous months and some with no time scale [6,27,28]. Similarly, the increased use of technology by young people and the lack of feedback may be two other reasons for this.

From the results, it can be determined that the behaviors that are most endured, performed or observed by the participants are:

- Sending offensive, insulting messages by mobile phone or over the internet;
- Anonymous calls aimed at frightening;
- Slanderous statements on the internet, character-smearing lies;
- Making offensive, insulting calls by mobile phone or over the internet;
- Blackmail or threats via messages or calls.

It is observed that the three most prevalent behaviors are repeated in the three roles, thus confirming our Hypothesis 3. This is consistent with other studies measuring various behaviors [11,13,30,31], which found that the cyberbullying assessed tended to coincide in the three roles involved. The data also confirm that of Ortega et al. [3] and Garaigordobil [13], who found that cyberspace can be a threatening and ugly world with few rules regarding socially acceptable behaviors. This information is important as it allows for the creation of prevention programs that focus more on those most commonly performed behaviors so that adolescents learn about the consequences.

Finally, the correlations between the roles show that cybervictims are more likely to act as cyberaggressors towards others and to play the role of bystander in other cases. The data therefore confirm our Hypothesis 4, but with caution, since significant and positive correlations are found between the different roles. Following the explanation of Garaigordobil [13] and Kowalski, Limber and Agatston [32], cybervictims may become cyberaggressors in order to feel powerful and superior, and so relieve the dejection and impotence resulting from victimization, or it may be due to cybervictims externalizing their feelings through cyberaggressive behaviors. However, the percentage reported by Kowalski and Limber was higher than that found in our study.

## 5. Conclusions

In conclusion, compared to other studies [33], the data found a situation in the Region of Murcia that is growing and cannot be ignored. The figures are becoming increasingly alarming, even if the percentages of severe cases are not very high. It should be noted that our study uses the same instrument as Garaigordobil [13]. Our findings are slightly higher, especially for cybervictims. This may be because more adolescents are using technology with increasing frequency [4], and without parental supervision, enhancing the disinhibition effect [34,35] used to describe the lowering of psychological restraints, which often serve to regulate behaviors in the online social environment.

The data collected should not remain mere data. The information is a clear call for psycho-pedagogical proposals to improve prevention and intervention in cases of cyberbullying and, hence, enhance coexistence and reduce the cases of school violence overall in the schools of the Region of Murcia. Cyberbullying is a social and public health issue, with devastating effects on victims both academically and in their professional future [36]. As reflected in other studies [37], teachers have an important role to play but are often unaware of how to tackle the problem. They need training in order to assist children to use ICT appropriately, particularly when we take into consideration the most vulnerable people who suffer the most from cyberbullying [38]. The idea is not to prohibit ICT in centers, but to teach the appropriate use of it. Therefore, the data collected here will be sent to centers and administrations to help establish educational steps to prevent future cases of cyberbullying, and to set up an intervention protocol to combat the reality of cyberbullying in our classrooms.

### Limitations and Future Research

Although the main objective of the work was to advance knowledge about cyberbullying in the Region of Murcia (Spain), we cannot forget that there are certain limitations. Although the sample used is representative of the region in which it was carried out, the results cannot be extrapolated to other parts of the country, so the scope of its applicability is somewhat reduced. Furthermore, it was not possible to establish a distinction between casual and frequent behaviors of the children involved in cyberbullying. This would require a qualitative cohort study to determine how many actual victims of cyberbullying there are, in addition to being able to conduct a more comprehensive analysis. Another limitation is related to the methodological design. The transversal research model prevents the determination of whether the cases of cyberbullying are a “blip” or are maintained over time. To do this, it would be necessary to perform a longitudinal study, as has been the case in other countries [39,40].

## Figures and Tables

**Table 1:** Frequency (F) and percentage of participants suffering, perpetrating or witnessing cyberbullying behaviors in the last year.

	Victims				Aggressors				Bystanders			
	Never	Sometimes	Often	Always	Never	Sometimes	Often	Always	Never	Sometimes	Often	Always
Items	*F* (%)	*F* (%)	*F* (%)	*F* (%)	*F* (%)	*F* (%)	*F* (%)	*F* (%)	*F* (%)	*F* (%)	*F* (%)	*F* (%)
1	692 (72.8)	218 (22.9)	27 (2.8)	13 (1.4)	831 (87.5)	110 (11.6)	6 (0.6)	3 (0.3)	522 (55.1)	302 (31.9)	107 (11.3)	17 (1.8)
2	794 (83.7)	124 (13.1)	27 (2.8)	4 (0.4)	895 (94.2)	50 (5.3)	4 (0.4)	1 (0.1)	613 (64.7)	251 (26.5)	73 (7.7)	11 (1.2)
3	924 (97.6)	18 (1.9)	3 (0.3)	2 (0.2)	939 (98.9)	10 (1.1)	0 (0)	0 (0)	766 (80.6)	119 (12.5)	46 (4.8)	13 (1.4)
4	897 (94.4)	44 (4.6)	6 (0.6)	3 (0.3)	916 (96.4)	30 (3.2)	2 (0.2)	2 (0.2)	670 (70.8)	206 (21.8)	57 (6)	13 (1.4)
5	917 (96.5)	28 (2.9)	1 (0.1)	4 (0.4)	934 (98.3)	15 (1.6)	0 (0)	1 (0.1)	754 (79.5)	139 (14.7)	43 (4.5)	12 (1.3)
6	738 (77.8)	165 (17.4)	36 (3.8)	10 (1.1)	862 (90.7)	77 (8.1)	8 (0.8)	3 (0.3)	639 (67.5)	211 (22.3)	75 (7.9)	22 (2.3)
7	837 (88.1)	92 (9.7)	15 (1.6)	6 (0.6)	916 (96.4)	30 (3.2)	4 (0.4)	0 (0)	727 (76.8)	156 (16.5)	48 (5.1)	16 (1.7)
8	911 (96.1)	29 (3.1)	5 (0.5)	3 (0.3)	944 (99.5)	3 (0.3)	2 (0.2)	0 (0)	870 (91.6)	56 (5.9)	13 (1.4)	6 (0.6)
9	904 (95.2)	36 (3.8)	8 (0.8)	2 (0.2)	941 (99.1)	8 (0.8)	1 (0.1)	0 (0)	818 (86.1)	91 (9.6)	35 (3.7)	3 (0.3)
10	842 (88.6)	96 (10.1)	10 (1.1)	2 (0.2)	933 (98.2)	16 (1.7)	1 (0.1)	0 (0)	756 (79.9)	132 (14)	49 (5.2)	9 (1)
11	927 (97.7)	19 (2)	1 (0.1)	2 (0.2)	940 (98.9)	9 (0.9)	0 (0)	1 (0.1)	751 (79.3)	141 (14.9)	41 (4.3)	14 (1.5)
12	895 (94.3)	42 (4.4)	9 (0.9)	3 (0.3)	940 (99.1)	8 (0.8)	1 (0.1)	0 (0)	774 (81.8)	126 (13.3)	32 (3.4)	14 (1.5)
13	913 (96.1)	34 (3.6)	3 (0.3)	0 (0)	939 (98.8)	10 (1.1)	1 (0.1)	0 (0)	821 (86.7)	93 (9.8)	24 (2.5)	9 (0.9)
14	911 (95.9)	29 (3.1)	9 (0.9)	1 (0.1)	939 (98.9)	8 (0.8)	2 (0.2)	0 (0)	852 (90.1)	68 (7.2)	22 (2.3)	4 (0.4)
15	784 (82.7)	119 (12.6)	33 (3.5)	12 (1.3)	932 (98.2)	14 (1.5)	3 (0.3)	0 (0)	679 (71.7)	162 (17.7)	68 (7.2)	38 (4)

Notes: items or behaviors: 1 = offensive/insulting text messages; 2 = offensive/insulting phone calls; 3 = bully, record and post on the internet; 4 = disseminate private photos/videos; 5 = take photos in changing rooms, at the beach ... to broadcast; 6 = anonymous threatening phones calls; 7 = blackmail; 8 = sexual harassment by mobile phone/internet; 9 = identity theft; 10 = password theft; 11 = manipulating photos/videos and dissemination of same; 12 = isolating on social networks; 13 = blackmailing without broadcasting intimacy; 14 = death threats; 15 = slandering and spreading discrediting rumors.

**Table 2:** Bivariate partial correlation, controlling for effect of sex and age on the total indexes of the four roles.

	1	2	3
1. Cybervictimization			
2. Cyberaggression	0.39 *		
3. Cyberbystander	0.53 *	0.38 *	
4. Cyberaggressive-cybervictim	0.94 *	0.68 *	0.56 *

Note: * *p* < 0.001.
